# Preparation of a multilayer antibacterial film and its application for controlling postharvest disease in temperate fruit (including apple, pear, and peach) under ambient storage

**DOI:** 10.1002/fsn3.3477

**Published:** 2023-07-05

**Authors:** Jingjing Hu, Wenxiao Jiao, Qingmin Chen, Bangdi Liu, Maorun Fu

**Affiliations:** ^1^ College of Food Science and Engineering Qilu University of Technology (Shandong Academy of Sciences) Jinan China; ^2^ College of Food Science and Engineering Shandong Agricultural and Engineering University Jinan China; ^3^ Academy of Agricultural Planning and Engineering Ministry of Agriculture and Rural Affairs Beijing China

**Keywords:** antibacterial film, carboxymethyl cellulose, chitosan, lemon essential oil, self‐assembled film, ε‐Polylysine

## Abstract

The objective of this study was to provide formulation of a new multilayer antibacterial film and to investigate the optimal use concentration of chitosan and carboxymethyl cellulose in the range from 0.5% to 2%, as well as its application for controlling postharvest disease in temperate fruit (apple, pear, and peach). The multilayer antibacterial film used chitosan (CS) and carboxymethyl cellulose (CMC) as polysaccharide macromolecule, lemon essential oil (LEO) as active agent, and ε‐polylysine (ε‐PL) as the main antibacterial ingredient. The results showed that the physical properties of the self‐assembled film were adjusted by the electrostatic layer‐by‐layer (LbL) deposition. Fourier transform infrared (FT‐IR) analysis and thermogravimetric (TGA) revealed that hydrogen bonds were generated during the self‐assembly of CS‐LEO/CMC‐ε‐PL film, resulting in changes in intermolecular interactions and thermal stability. Furthermore, compared with CS‐LEO single‐layer film, the multilayer film exhibited higher retention rate of LEO. In vivo test, the self‐assembled film significantly inhibited the infection of postharvest pathogenic fungi including *Penicillium expansum* (*P. expansum*) and *Alternaria alternata* (*A. alternata*) on fruit. To summarize, the CS‐LEO/CMC‐ε‐PL LbL self‐assembly coating notably controlled postharvest pathogen rot on fruit, and reduced the loss of fruit during storage and transportation. Our results suggest that the polysaccharide‐based edible coating prepared in this work may offer an alternative to synthetic waxes.

## Graphical abstract

The CS‐LEO/CMC‐e‐PL LbL self‐assembly coating notably controlled postharvest pathogen rot on fruit, and reduced the loss of fruit during storage and transportation. Our results suggest that the polysaccharide‐based edible coating prepared in this work may offer an alternative to synthetic waxes.

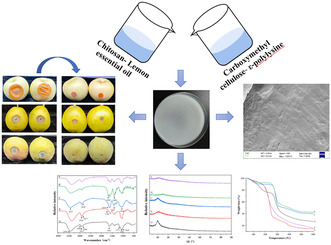

## 1 INTRODUCTION

The nutritive value and original taste, color, and appearance of the fruit are largely modified when the environmental conditions are unfavorable after the harvest, and even worse, it can undergo enzymatic and nonenzymatic browning, odor, discoloration, shrinkage, surface hardening, etc. (Elik et al., 2019). During storage, fruit that is rich in moisture and nutrients could be an ideal substrate for microorganism development, so they may rot and must be discarded with substantial losses (Arnon‐Rips & Poverenov, 2018). At present, the postharvest preservation methods of fruit mainly consist of low storage temperatures, modified atmospheres, fungicides treatment, and edible coatings (Prasad, Das, Maurya, Jain, & Dwivedy, 2022). On account of the possibility of reduced fruit quality caused by low storage temperatures, different gas formulations for per product type, or potential adverse health effects of conventional fungicides, edible coatings are widely considered due to its convenience and low priced (Zhang et al., 2020). Edible film using biodegradable polysaccharides, proteins and lipids as the main material, edible polyol as plasticizer, through certain processing procedures. Among most film matrices, polysaccharide films have been widely studied due to their good biocompatibility (Hassan et al., 2018).

Lately, a growing number attention has been concentrated on polysaccharide‐based edible coatings, especially chitosan and cellulose derivatives. Chitosan (CS) is a cationic polymer, and its antibacterial and gas barrier properties have significant advantages in edible coating (An et al., 2018). Chitosan still has many shortcomings when used alone, such as its solubility, moisture retention, viscosity, tensile strength, and antioxidant capacity (Kerch, 2015). Adding essential oil can improve the physical and chemical properties of chitosan, but cannot change its shortcomings of high brittleness and high water solubility (Lee et al., 2018; Zhang et al., 2021). Other natural macromolecules (polysaccharides, proteins, and lipids) with different properties can be combined with the chitosan‐essential oil base film in order to solve the shortcomings of chitosan‐essential oil film by layer‐by‐layer (LbL) approach (Marand et al., 2023). The layer‐by‐layer (LbL) approach significantly controls the physicochemical property of edible coatings by exploiting the charge interactions between molecules of charged materials. (Arnon‐Rips & Poverenov, 2018). Electrostatic layer‐by‐layer deposition is the continuous cross‐linking and electrostatic interaction of two solutions with opposite charges to form an electrostatically stable film at room temperature (Huang et al., 2021). Carboxymethyl cellulose (CMC) is an excellent anion linear polymer with safety, biodegradability, and good film‐processing properties (Nazoori et al., 2023). Chen et al. (2020) reported that LbL coating assembled by CS and CMC notably improved the physiological attributes of lemon fruit. However, there has been no report on the LbL self‐assembled coating films of CS‐CMC with antibacterial active ingredients added. In this paper, the CMC‐ε‐PL of oppositely charged was self‐assembled with the CS‐LEO single layer, so as to reform the properties of the coating material.

This study aims to develop multilayer antimicrobial edible coating incorporating active agents by electrostatic deposition techniques, further improving the retention rate of lemon essential oil. The physicochemical characteristics were also assessed in the research. Moreover, the research also demonstrated the application for controlling postharvest disease in fruit.

## 2 MATERIALS AND METHODS

### 2.1 Materials

Lemon essential oil (LEO) was purchased from Fangdai Cosmetics Co., Ltd. Chitosan, degree of deacetylation >95% and viscosity 20–500 map, s (1%, 20°C), was acquired from Walson Biotechnology Co., Ltd. Carboxymethyl cellulose (CMC) was purchased from Beijing Coollaber Technology Co., Ltd. ε‐Polylysine (ε‐PL) applied in the work was obtained from Zhengzhou Bainafo Bioengineering Co., Ltd., and the purity was 99%. All other chemical reagents used in this work were of analytical grade.

#### 2.1.1 Fruit and fungi

A total of 150 commercially mature fresh fruits for each variety, including ‘Golden delicious’ apple, ‘Qingzhou’ peach, and ‘Korla fragrant’ pear, were purchased from a local Changqing Supermarket in Jinan, Shandong Province in China, and experimental processing was started immediately. Fruit of uniform size and free of damage and diseases were selected for inoculation with the pathogen, fully immersed in 0.05% (v/v) concentration of sodium hypochlorite solution for 2 min, and then air‐dried at room temperature. *Penicillium expansum* (*P. expansum*) and *Alternaria alternata* (*A. alternata*) were cultured for 5 days, respectively, then the spore suspension was collected after filtration. After filtering through four layers of sterile gauze, a spore suspension without any adhering mycelia was collected for determining spore concentration with the aid of a hemocytometer. Subsequently, the initial concentration of spore suspension was adjusted to 1 × 10^6^ spores mL^−1^ with sterile water.

### 2.2 Preparation of CS‐LEO/CMC‐ε‐PL

Chitosan solution (1.0%, w/v) was dissolved in the acetic acid solution (1.0%, v/v). CMC powder (1.0%, w/v) was dissolved in sterilized water and stirred until completely dissolved. Tween‐80 at the concentration of 0.2% (w/v) was added as an emulsifier and glycerol at the concentration of 0.1% (w/v) as a plasticizer to CS solution, and then stirred for half an hour. The CS‐LEO/CMC‐ε‐PL LbL film was assembled by 1.0% CS and 1.0% CMC solution, which put LEO (1.5%, v/v) in CS solution and put ε‐PL powder (1.0%, w/v) in CMC solution, respectively.

All film‐forming solutions were ultrasonicated (240 W, 40 KHz) for 30 min for degassing treatment. The 25‐mL CS, CMC, CS‐LEO, and CMC‐ε‐PL solutions were separately cast onto Teflon mold (side length of 9 cm), then dried at 40°C for 8 h in a drying oven to form pure CS, CMC, CS‐LEO, and CMC‐ε‐PL single layer film. Then, 25 mL of 1% (v/v) CMC‐ε‐PL solution was cast onto top of the CS‐LEO layer as the second layer, and the bilayer was dried in a drying oven at 45°C for 8 h to obtain CS‐LEO/CMC‐ε‐PL LbL self‐assembly film. All films developed were stripped carefully and preserved in a desiccator for further usage.

### 2.3 Physical properties

#### 2.3.1 Film thickness

The film thickness was measured using a digital micrometer (Mitutoyo Measuring Instruments Co., Ltd.) with an accuracy of 0.001 mm. Six different positions were measured for each film. Each sample was treated with 10 replications.

#### 2.3.2 Transparency

The method for measuring transparency was based on Zhang et al. (2019) with few modifications. Measuring the thickness of rectangular films and absorbance at 600 nm, and using the formula to get the transparency of films. The thickness of the rectangular film and the absorbance at 600 nm were measured, and the transparency of the film was assessed by the following Equation (1). Each sample was treated with 10 replications.

(1) $$\text{Opacity}\;\left(A\;{\mathrm{mm}}^{-1}\right)=\frac{A}{x}$$

where *A* is the absorbance value at 600 nm and *x* stands for the thickness of the film (mm).

#### 2.3.3 Moisture content

The determination of moisture content referred to the previous reports with slight changes (Ojagh et al., 2010). Film samples were dried to a constant weight at 105°C. Moisture content was calculated according to the following formula (2). The initial weight *M*_*i*_ (g) and dried weight *M*_*d*_ (g) of the film samples were recorded.

(2) $$\text{Moisture contents}\;\left(\%\right)=\frac{M_{i}-M_{d}}{M_{i}}\times 100$$

#### 2.3.4 Water vapor permeability (WVP)

The determination of WVP was carried out according to changes of original mass (Binsi et al., 2013). The film samples were cut into squares, weighed to record the original mass, and used to seal a weighing cup (4 cm diameter and 4 cm depth) with 3 g of CaCl_2_. The cup was put into a desiccator with saturated potassium iodide (20°C, 70% relative humidity). The cup was weighed every 6 h for 2 days until it reaches a constant weight. The water vapor permeability of samples was calculated according to the formula described later (3). Each sample was treated with three replications.

(3) $$\text{Water vapor permeability}=\frac{M\times x}{t\times A\times \mathrm{\Delta p}}$$

where *M* stands for the weight difference (g), *x* stands for the film thickness (m), *t* represents the permeation time (s), *A* is the film permeation area (m^2^), and *Δp* stands for the difference of permeation area vapor pressure (2339 Pa at 20°C).

#### 2.3.5 Mechanical properties

The determination of mechanical properties for films was developed according to the previous method (Beak et al., 2018; Shah et al., 2016) with slight modifications. Film samples were separately cast onto Teflon mold into rectangular shape of 1.5 × 10 cm to determine the tensile strength (TS) and elongation at break (E). The values of TS and E for film samples were evaluated with the help of a texture analyzer (XLW [PC], Languang Electromechanical Technology Co., Ltd). The final effective length of the stretching was 70 mm, and the stretching rate was 50 mm/min. Tensile strength and elongation at break of films were evaluated according to the following Equations (4) and (5). Each film was measured at least five times. Each sample was conducted for three repetitions.

(4) $$\text{Tensile strength}\;\left(\mathrm{MPa}\right)=\frac{F}{x\times w}$$

(5) $$\text{Elongation}\;\mathrm{at}\;\text{break}\;\left(\%\right)=\frac{\mathrm{\Delta x}}{x_{0}}$$

where *F* (N) represents the maximum force at rupture of the film, *x* (mm) represents the film thickness, and *W* (mm) stands for the width of film piece; *x*_*0*_ (mm) stands for the initial length of film piece, and *Δx* (mm) is the increase in length.

### 2.4 Characterization of films

#### 2.4.1 Morphology by scanning electron microscopy (SEM)

The film samples were obtained in squares (0.5 × 0.5 cm^2^) to observe the modification characteristics of film surface. Microscopic morphology of films under the magnification of 5000× was observed by scanning electron microscope with the accelerating voltage of 5 kV (SUPRA 55, Carl Zeiss AG).

#### 2.4.2 Fourier transform infrared (FT‐IR) analysis

The thin film samples were lyophilized in a vacuum freeze dryer (TS6003), ground into powder in liquid nitrogen, and stored under dry conditions for later use. The lyophilized samples were taken to mix with desiccated potassium bromide (KBr) powder at a ratio of 1:100, then ground and pressed into tablets. The peaks were scanned and collected in the range of 4000–500 cm^−1^, and the difference of each peak was observed.

#### 2.4.3 Thermogravimetric analysis (TGA)

Thermogravimetric analysis (TGA) of the films was conducted with the help of a thermogravimetric analyzer (Perkin Elmer Pyris 1). The pretreatment of samples was still ground into powder after lyophilized. The sample was placed in a ceramic crucible and heated from 30°C to 600°C under a flowing nitrogen atmosphere.

#### 2.4.4 X‐ray diffraction analysis (XRD)

The measurement of wide‐angle X‐ray diffraction was performed using an In situ X‐ray diffractometer (X'Pert3 Powder, PANalytical B.V). The samples were ground into powder after lyophilized, then flattened them out in the sample cell. The patterns were recorded in the region of 2θ from 10° to 90°.

### 2.5 Release and retention properties of LEO in film

#### 2.5.1 Determination of standard curve

Certain concentrations of lemon essential oil solutions were prepared by using anhydrous ethanol. Scanning the above solution with an ultraviolet–visible–near‐infrared spectrophotometer to determine that its maximum absorption wavelength is 332 nm. At its maximum absorption wavelength, standard solutions with a series of concentration gradients were prepared, while anhydrous ethanol was used as blank control group. Standard curve of lemon essential oil was obtained by linear regression of absorbance to concentration.

#### 2.5.2 Determination of LEO concentration

The LEO concentration in CS‐LEO film and LbL self‐assemble film were observed according to the reports by Zhang et al. (2019) with slight modifications. The film was punched into regular small disks (6 mm) by punchers to make sure that the disk film had the same area and was stored at room temperature. Ten disks were placed at a time in 2 mL of distilled water and 5 mL of anhydrous ethanol, and finally 1 mL of acetic acid in order to better dissolve the film to determine the LEO content. The mixture was centrifuged after shaking (120 rpm) at 25°C for 24 h. The absorbance value of the supernatant was measured using ultraviolet and visible spectrophotometer (UV‐9000, METASH) at 332 nm. The LEO concentration was quantified using a standard curve corrected from the known concentration of LEO anhydrous ethanol solution.

### 2.6 The effect of the layer‐by‐layer coatings in fruit

In this work, fruits were randomly divided into five groups (A1, A2, A3, A4, and A5) of 10 fruit each, with three replicates for each group. A 1.5% (v/v) LEO was selected to be incorporated into the CS solution to arrange a CS‐LEO coating solution. The concentration of each group is as follows. A1 was treated with sterile water. A2 was 0.5% chitosan (w/v), 1.5% (v/v) LEO, 0.2% (w/v) tween 80, 1.0% (w/v) glycerol mixed solution as the first film, 0.5% carboxymethyl cellulose (w/v), and 1.0% ε‐polylysine (w/v) mixed solution as the second film. Both concentrations of chitosan (w/v) and carboxymethyl cellulose (w/v) were adjusted to 1.0%, 1.5%, or 2% in A3, A4, or A5 groups, respectively. The postharvest fruit was soaked in the coating solution of CS‐LEO, taken out, drained, and then air‐dried to obtain a single‐layer coated fruit. Then, the coated fruit were soaked in the coating solution of CMC‐ε‐PL for 1–2 min, then air‐dried to obtain double‐layer coated fruit.

Effect of LbL films on fruit was performed referring to Jiao et al. (2020) and Moussa et al. (2013) with slight modifications. According to the main pathogenic bacteria that caused fruit rot, spore suspensions of different genera were inoculated on different fruit. In this study, pears were inoculated with *A. alternata*, apples were inoculated with *P. expansum*, and peaches were inoculated with *P. expansum*. A sterile stainless steel needle was used to puncture the fruit on both sides of the equator of each fruit to form two symmetrical and uniform holes. Each wound of all fruit was inoculated with 10 μL of spore suspension at 1 × 10^6^ spores mL^−1^ and then air‐dried at room temperature in a plastic frame and packed in a polyethylene bag with small holes. 90%–95% relative humidity (RH) was maintained in plastic frames with wet gauze and stored at 25°C to develop disease. After 7 days, the disease condition of fruit was observed and lesion diameters were recorded by the cross method. The following formula (6) was used to calculate the lesion diameter. The experiment was performed three times.

(6) $$\text{Lesion diameter}=\frac{\sum \text{Lesion diameter of infected fruit}}{\text{Total number of fruit in each treament}}$$

## 3 RESULTS AND DISCUSSION

### 3.1 Film thickness

The physical and mechanical properties of the films were tested as in Table 1. The strength of respiration is determined by the thickness. The test results proved that adding LEO has no great influence on the thickness of CS film. After adding ε‐PL to the CMC film, the thickness of the CMC‐ε‐PL film was significantly increased (He et al., 2021). But after self‐assembly of CS‐LEO and CMC‐ε‐PL, the thickness of the CS‐LEO/CMC‐ε‐PL film was obviously lower than the sum of the two. The reduction of film thickness was beneficial to fruit respiration.

**TABLE 1:** Thicknesses, moisture contents, opacity, water vapor permeability, and mechanical properties of CS, CMC, CS‐LEO, CMC‐ε‐PL, and CS‐LEO/CMC‐ε‐PL films.

Films	Thickness (μm)	Moisture Contents (%)	Opacity (A mm^−1^)	Tensile Strength (MPa)	Elongation at Break (%)	Water vapor permeability (10^−5^ g m^−1^ min^−1^ Pa^−1^)
CS	59.13 ± 3.28^c^	43.39 ± 0.84^a^	7.34 ± 2.25^b^	7.61 ± 2.75^a^	1.72 ± 0.36^a^	3.06 ± 0.28^c^
CMC	40.50 ± 2.01^d^	33.55 ± 2.42^b^	11.04 ± 0.77^a^	2.89 ± 0.47^bc^	1.74 ± 0.34^a^	2.58 ± 0.09^c^
CS‐LEO	60.17 ± 0.78^c^	42.20 ± 1.00^a^	8.79 ± 0.51^b^	0.62 ± 1.01^ab^	1.69 ± 0.23^a^	4.81 ± 0.15^b^
CMC‐PL	117.83 ± 11.61^b^	15.32 ± 0.47^c^	4.16 ± 0.48^d^	1.25 ± 0.68^abc^	1.17 ± 0.06^a^	4.44 ± 0.32^b^
CS‐LEO/CMC‐PL	138.42 ± 7.60^a^	16.30 ± 0.95^c^	5.82 ± 0.14^c^	2.56 ± 0.44^c^	1.21 ± 0.01^a^	6.88 ± 0.27^a^

*Note*: Values followed by the same letter in the column on the same day were not significantly different according to Duncan's multiple range test (*p* < .05). Data are accompanied by standard errors of the means (*n* ≥ 3).

### 3.2 Transparency

The opacity of CMC‐ε‐PL film was significantly lower compared to pure CMC film (*p* < .05), which significantly affected the food appearance (He et al., 2020). However, when CS‐LEO and CMC‐ε‐PL films were self‐assembled, the CS‐LEO/CMC‐ε‐PL film slightly increased the opacity to enhance the light barrier and reduced the oxidative deterioration of fruit and vegetables (Odjo et al., 2022). The phenomenon may also be related to light scattering due to the different refractive indices of lipid droplets distributed throughout the film network (Sánchez‐González et al., 2009).

### 3.3 Mechanical properties

The incorporation of the LEO dispersed phase leads to a decrease in tensile strength and elongation at break, which makes the film softer and less resistant to break (Jiang et al., 2020). The weak mechanical response was mainly due to discontinuities in the polymer matrix caused by the incorporation of LEO, or polymer chain interactions when the oil component was present (Vargas et al., 2009). Similarly, it could also be explained why the addition of ε‐PL makes the tensile strength and elongation at break smaller combined with CMC. However, when CS and CMC were self‐assembled, the tensile strength of the layer‐by‐layer film was better than the sum of CS film and CMC film. It was the same as the result of CS‐CMC composited film (He et al., 2021).

### 3.4 Moisture content and water vapor permeability

For the most part, the moisture content of CS‐LEO decreased due to the addition of essential oils (Grande‐Tovar et al., 2018). The same phenomenon can be observed in this experiment. After adding ε‐PL, the moisture content of CMC‐ε‐PL was significantly reduced compared to monolayer CMC film. In this experiment, it was observed that the water vapor permeability was related to amount of added substances in the membrane. The highest water vapor permeability was the CS‐LEO/CMC‐ε‐PL film, while CS and CMC film had the lowest performance.

In spite of the greater thickness, the CS‐LEO/CMC‐ε‐PL self‐assembly film was significantly (2.24 times) greater than that of pure CS film (*p* < .05) in the water vapor permeability, in agreement with their higher hydration level. CS‐LEO/CMC‐ε‐PL self‐assembled membranes form a more loosely packed porous matrix due to the swelling properties of the chitosan (Chen et al., 2020), thereby facilitating the water transport in the assembled structure.

### 3.5 Fourier transform infrared (FT‐IR) analysis

**FIGURE 1:**
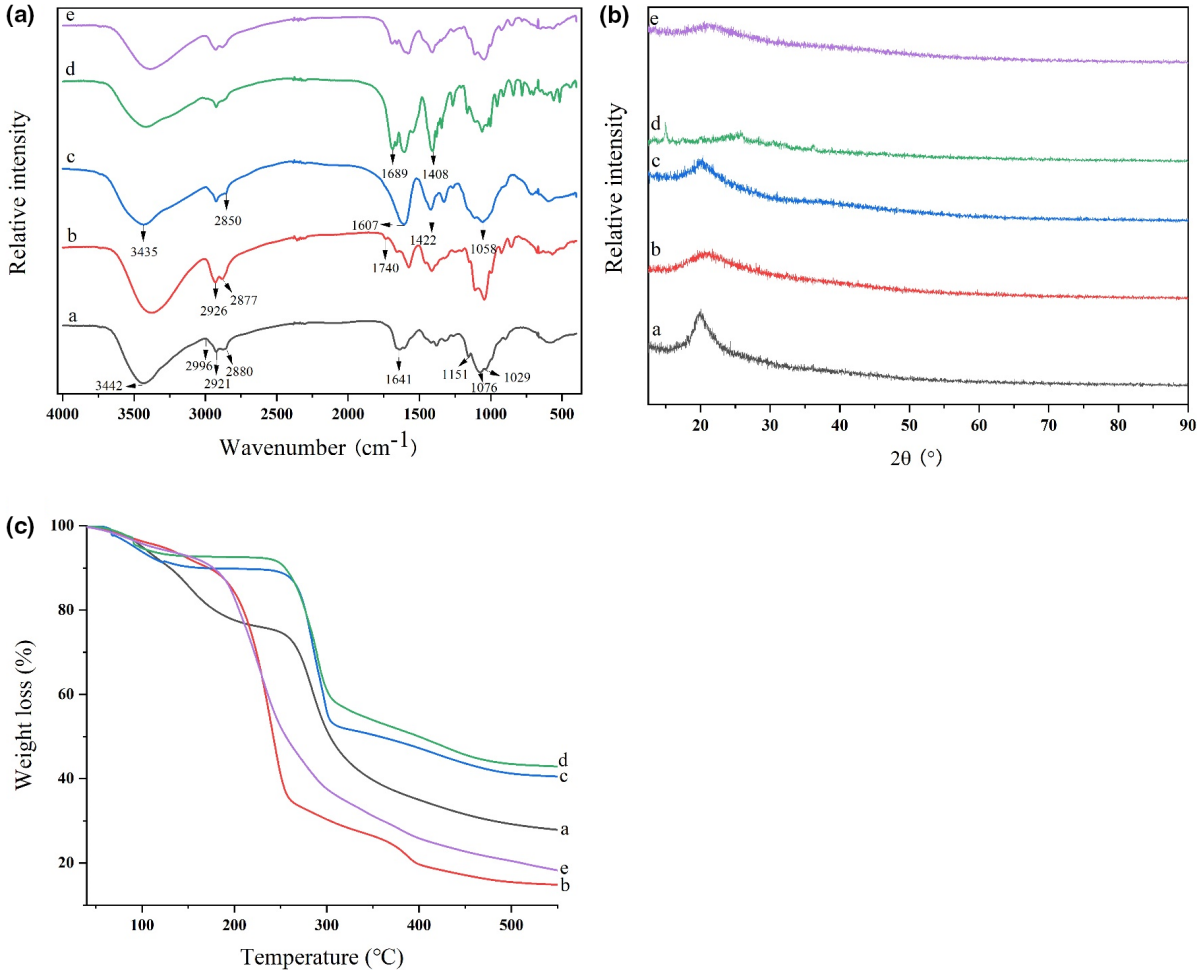
FT‐IR spectra (a), X‐ray diffraction pattern (b), thermogravimetric curves (c) of CS single layer film (a), CS‐LEO single layer film (b), CMC film (c), CMC‐ε‐PL film (d), and CS‐LEO/CMC‐ε‐PL self‐assembly film (e).

The result of FT‐IR is shown in Figure 1a. The broad peak of the CS film appearing at 3442 cm^−1^ corresponded to the overlapping stretching vibration of O‐H and N‐H (Kundu et al., 2020). A distinctive absorption band at 1641 cm^−1^ was assigned to C=O stretching of amide group (amide I) (Soltanzadeh et al., 2021). Compared with ordinary chitosan film, CS‐LEO film exhibits different FT‐IR spectra. With the incorporation of the LEO, the peak around 2921 and 2880 cm^−1^ was bathochromic shifting to 2926 and 2877 cm^−1^ and became stronger (Zhang et al., 2019), which might be related to the C‐H stretching (‐CH_2_) of the LEO (Bagheri et al., 2021). In addition, the new absorption peak appeared at 1740 cm^−1^, indicating the formation of ester bond between CS and LEO molecules (Kadam et al., 2018; Liu et al., 2017).

The stretching vibrations of the carboxylic acid groups of CMC film produced peaks at 1422 and 1607 cm^−1^. The peak at 1058 cm^−1^ of the CMC film is associated with the asymmetric stretching vibrational band of the ether group (He et al., 2020). Many new peaks appeared in the spectrum of CMC‐ε‐PL after blending with ε‐PL, and the peaks shifted from 1607 cm^−1^, 1422 cm^−1^ to 1689 cm^−1^, 1408 cm^−1^. Interaction between ε‐PL and CMC matrix might shift the absorption peak. The spectra in the CS‐LEO/CMC‐ε‐PL films were found to contain characteristic bands of CS and CMC from Figure 1a. The band at 3400–3281 cm^−1^ widened as the CMC content increased (Jia et al., 2022), indicating intermolecular hydrogen bonding between CS and CMC in the films. When CMC was added, more carboxylate anions were drawn into the CS film, causing the peak of C=O at 1641 cm^−1^ to be enhanced and shifting to a lower wave number.

### 3.6 X‐ray diffraction (XRD) analysis

Figure 1b demonstrates the X‐ray diffraction (XRD) spectra of each film. Diffraction spectrum of pure CS film showed a sharp peak at 2θ of 22.7°, which was attributed to its high degree of crystallinity (Tan et al., 2015). As seen in the XRD pattern of CS‐LEO, the inclusion of LEO resulted in a broad peak which proved the complex change had been accomplished (Prasad, Das, Maurya, Jain, & Dwivedy, 2022). Due to the addition of ε‐PL, the patterns of the CMC‐ε‐PL blended film showed wider and more intense peaks, indicating their semicrystalline nature. The peak intensities of the CS‐LEO/CMC‐ε‐PL self‐assembled film were lower and broader than that of the CMC and CS film, which might be attributed to the intermolecular hydrogen bonding.

### 3.7 Thermogravimetric analysis (TGA)

TGA (Figure 1c) displays the connection between mass loss and temperature change. Two main steps of weight loss are shown in the thermograms. The first stage is before the temperature reaches 150°C (Chu et al., 2019). As shown in Figure 1c, the TGA curve of the CMC film had the same trend as the CMC‐ε‐PL film. The second stage is the rapid thermal degradation of the polymer at 180–340°C. The supplement of LEO immensely changed the weight loss trend of the CS. The TGA curve of CS‐LEO/CMC‐ε‐PL was very similar but slightly different from CS‐LEO. A large weight loss phase could be detected at 200–300°C in the curves of both films. However, when CS‐LEO and CMC‐ε‐PL were assembled, the thermal stability of the LbL film increased, which was related to the formation of hydrogen bonds after self‐assembly.

### 3.8 The release properties of LEO

**FIGURE 2:**
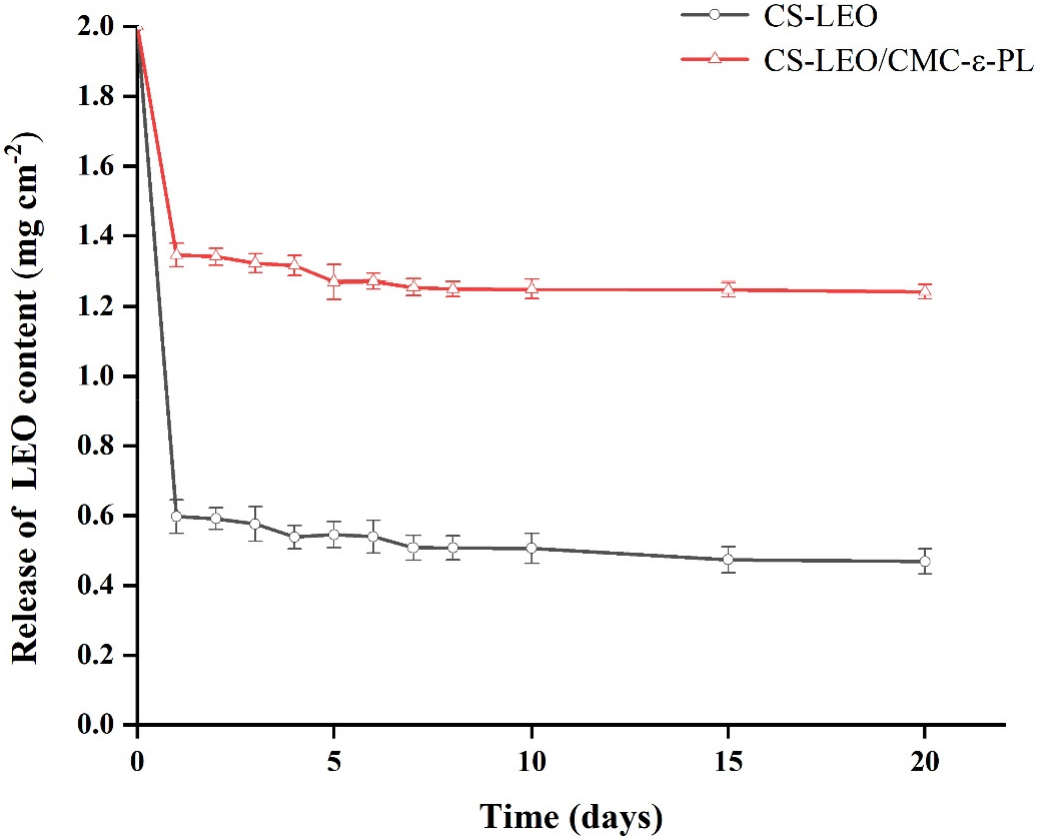
The release properties of LEO in composite film. The vertical bars indicate the standard deviations for each treatment (*n* ≥ 3).

Essential oils are effective in delaying fungal decay in fruit, but their volatility is a significant disadvantage. The main benefit of the incorporation of LEO into CS is the reduction of their diffusion rate, which is beneficial to the shelf life of the food in practical application. Figure 2 shows the influence of layer‐by‐layer method on the release properties of LEO. The lemon essential oil in the self‐assembled film was released in large quantities on the first day. However, the release rate of CS‐LEO film was significantly higher than that of CS‐LEO/CMC‐ε‐PL film. But after the second day, the release amount bitty decreased.

It could also be observed that the release rate of LEO showed a stable trend after 2 days, and the retention effect of CS‐LEO/CMC‐ε‐PL film was 2.65 times higher than that of the CS‐LEO film, achieving the effect of embedding. It has been reported that CS and LEO molecules were mainly bonded through weak hydrogen bonds, and the LEO molecules in CS film were difficult to retain due to the hydrophilic CS and hydrophobic LEO (Chen et al., 2016; Hromiš et al., 2015; Shojaee‐Aliabadi et al., 2014). However, when LEO was self‐assembled layer by layer, the retention efficiency of LEO increased due to the formation of hydrogen bonds, which was consistent with our previous FT‐IR results. Therefore, our results showed that the layer‐by‐layer approach improved the LEO retention properties in multilayer films.

### 3.9 Film morphology by scanning electron microscopy (SEM)

**FIGURE 3:**
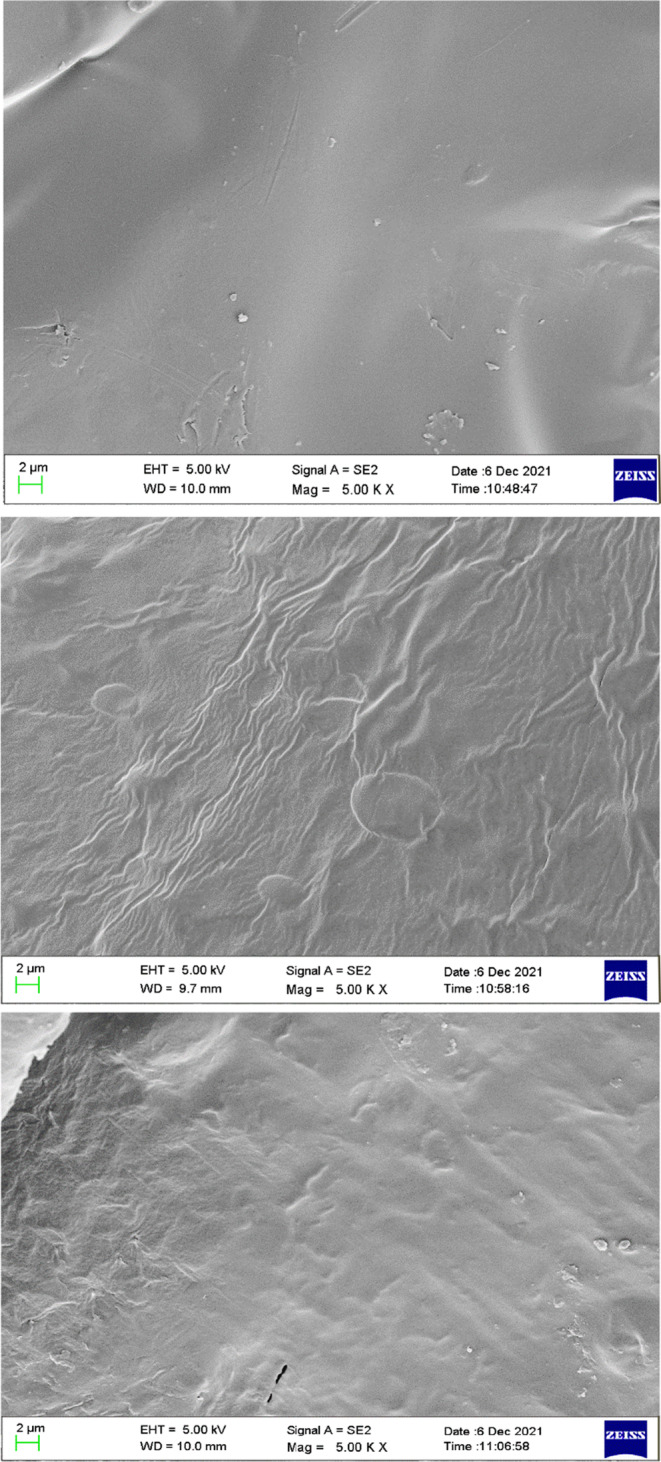
SEM micrographs of the surface of CS single‐layer film (a), CS‐LEO single layer film (b), and CS‐LEO/CMC‐ε‐PL self‐assembly film (c).

The antibacterial inclusion compound had a certain influence on the surface smoothness of the film. The neat CS films had a normal, smooth, and compact surface structure without pores or cracks (Peng & Li, 2014). The addition of LEO increased the roughness of the CS matrix as in Figure 3. Wrinkles appeared on the surface of CS‐LEO films, which might be attributed to the interaction between oil droplets and polysaccharide network. It suggested that flocculation and agglomeration occur during the film drying process. This result was also observed by Hafsa et al. (2016). After LbL self‐assembly of CS‐LEO film with CMC‐ε‐PL, this wrinkled surface disappeared, forming a more compact and continuous surface, but not as smooth as pure CS film surface, which might not be completely miscible with CMC and CS related (El‐Hefian et al., 2010).

### 3.10 The effect of the layer‐by‐layer coatings in fruit

**FIGURE 4:**
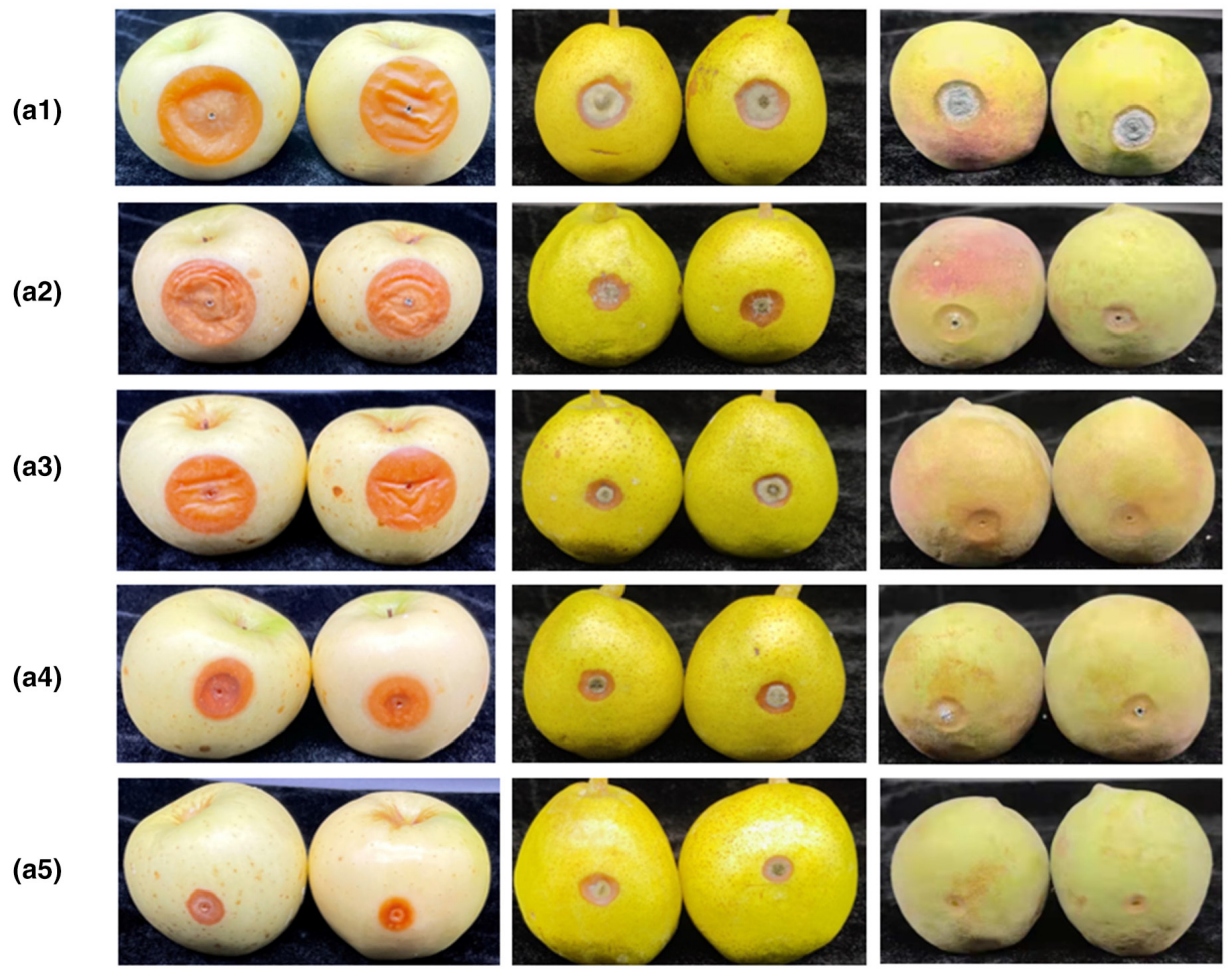
Appearance of apples, pears, and peaches after inoculated with *Penicillium. expansum*, *Alternaria. alternata*, and *Penicillium. expansum* for 6 days, respectively.

The CS‐LEO/CMC‐ε‐PL antibacterial film was applied to the postharvest preservation of apple, peach, and pear fruit. The layer‐by‐layer self‐assembled film has a significant inhibitory effect on fruit decay caused by the main postharvest pathogens such as *P. expansum*, *A. alternata*, and *P. expansum* (*p* < .05) (Figure 4).

**FIGURE 5:**
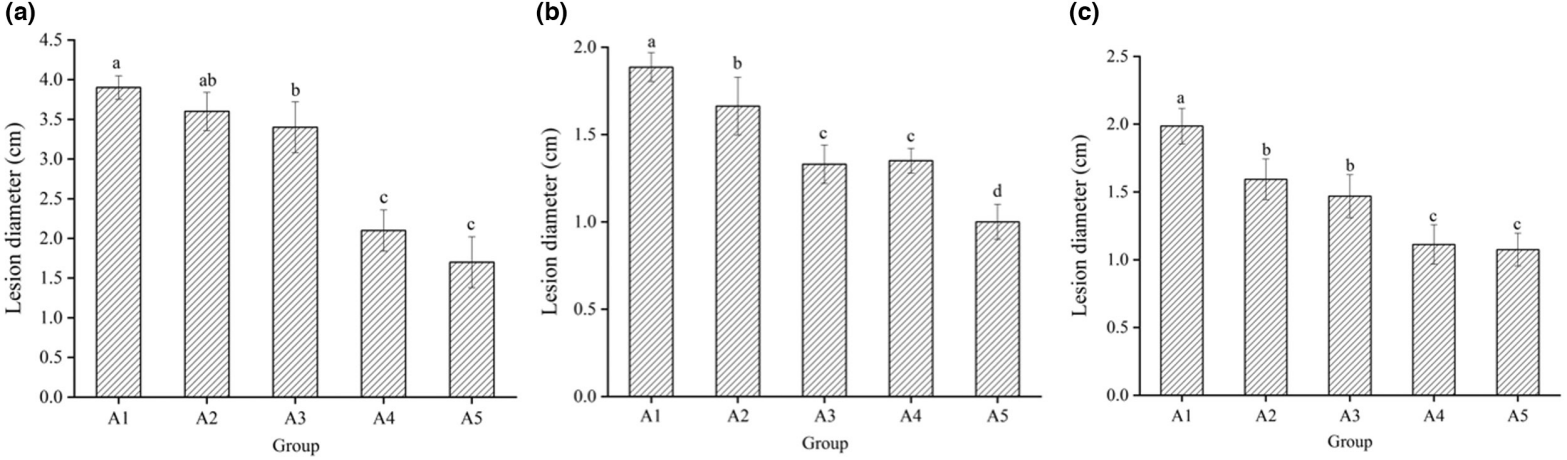
The lesion diameter of apple (a), pear (b), and peach (c) inoculated with *Penicillium. expansum*, *Alternaria. alternata*, and *Penicillium. expansum* with different coatings after storage. The vertical bars indicate the standard deviations for each treatment (*n* ≥ 3). Bars with different letters represent statistical differences (*p* < .05).

When the treatment groups were treated with groups of A1, A2, A3, A4, and A5, respectively, the diameters of pear lesions were reduced by 18.8%, 19.6%, 21.4%, 28.5% compared to the control group (Figure 5). Apples were treated with *P. expansum* at 25°C for 6 days. When doing the same deal with pears, the diameters of apple lesions were reduced by 38.1%, 33.3%, 42.9%, and 66.7% compared with the control group. Peaches were treated with *P. expansum* at 25°C for 4 days. Under the same treatment, the diameters of peach lesions were reduced by 27.3%, 26.0%, 44%, and 45.9% compared with the control group. However, when the concentration was higher than 1.5%, the film surface showed bad properties.

## 4 CONCLUSION

In this research, the multilayer antibacterial films based on CS and CMC as coating matrix and LEO and ε‐PL as main antibacterial active compounds were developed. The optimum concentration of substances on different fruits is different. In this article, considering lesion diameter and fruit quality, 1.5% (w/v) of CS and 1.5% (v/v) of LEO mixed solution, with 1.5% (w/v) of CMC and 1.0% (w/v) ε‐PL mixed solution at room temperature by LbL self‐assembly to form a film provided better protection to fruits. The composite antibacterial film was applied to the postharvest preservation of apple, peach, and pear fruit, and had a significant inhibitory effect on fungi decay caused by major postharvest pathogens such as *P. expansum*, and *A. alternata*. The self‐assembled film adjusted the thickness of the double‐layer film by means of electrostatic deposition, so that the thickness of the composite antibacterial film was much lower than the sum of the thicknesses of the coating CS‐LEO and CMC‐ε‐PL, which was beneficial to the respiration of fruit products. Hydrogen bonds were formed between CS and CMC after self‐assembly, which was further supported by the results of FT‐IR, TGA, and X‐ray diffraction analyses. The dense and continuous surface of the CS layer film observed by scanning electron microscopy confirmed the uniform distribution of LEO in the self‐assembled composite film. Compared with the CS‐LEO film, the CS‐LEO/CMC‐ε‐PL layer‐by‐layer self‐assembled film had a higher retention rate for LEO. The CS‐LEO/CMC‐ε‐PL LbL self‐assembly method realized a slow release of antibacterial active ingredients, effectively controlled fungal diseases, and greatly improved the control effect of a single chitosan coating on postharvest fruit diseases. In summary, the self‐assembly film system based on CS and CMC is a promising controlled release system for loading lemon essential oil, which could improve the efficiency of lemon essential oil in practical applications.

## AUTHOR CONTRIBUTIONS

**Jingjing Hu:** Investigation (equal); validation (equal); writing – original draft (equal). **Wenxiao Jiao:** Conceptualization (equal); methodology (equal); supervision (equal); validation (equal). **Qingmin Chen:** Writing – review and editing (equal). **Bangdi Liu:** Data curation (equal); validation (equal). **Maorun Fu:** Conceptualization (equal); methodology (equal); supervision (equal).

## CONFLICT OF INTEREST STATEMENT

We declare that we do not have any commercial or associative interest that represents a conflict of interest in connection with the work submitted.

## ETHICS STATEMENT

Ethics approval was not required for this research.

## Data Availability

The data are not shared, however, can be provided on request to the corresponding author.
